# Inverse kinetic isotope effects in the charge transfer reactions of ammonia with rare gas ions†

**DOI:** 10.1039/d1sc01652k

**Published:** 2021-06-22

**Authors:** A. Tsikritea, K. Park, P. Bertier, J. Loreau, T. P. Softley, B. R. Heazlewood

**Affiliations:** Department of Chemistry, University of Oxford, Physical and Theoretical Chemistry South Parks Road Oxford OX1 3QZ UK; Department of Physics, University of Liverpool Liverpool L69 7ZE UK b.r.heazlewood@liverpool.ac.uk; KU Leuven, Department of Chemistry Celestijnenlaan 200F B-3001 Leuven Belgium; School of Chemistry, University of Birmingham Edgbaston B15 2TT UK

## Abstract

In the absence of experimental data, models of complex chemical environments rely on predicted reaction properties. Astrochemistry models, for example, typically adopt variants of capture theory to estimate the reactivity of ionic species present in interstellar environments. In this work, we examine astrochemically-relevant charge transfer reactions between two isotopologues of ammonia, NH_3_ and ND_3_, and two rare gas ions, Kr^+^ and Ar^+^. An inverse kinetic isotope effect is observed; ND_3_ reacts faster than NH_3_. Combining these results with findings from an earlier study on Xe^+^ (Petralia *et al.*, *Nat. Commun.*, 2020, 11, 1), we note that the magnitude of the kinetic isotope effect shows a dependence on the identity of the rare gas ion. Capture theory models consistently overestimate the reaction rate coefficients and cannot account for the observed inverse kinetic isotope effects. In all three cases, the reactant and product potential energy surfaces, constructed from high-level *ab initio* calculations, do not exhibit any energetically-accessible crossing points. Aided by a one-dimensional quantum-mechanical model, we propose a possible explanation for the presence of inverse kinetic isotope effects in these charge transfer reaction systems.

## Abstract 2

Inverse kinetic isotope effects are observed in the charge transfer reactions of rare gas ions with ammonia molecules.

## Graphical abstract

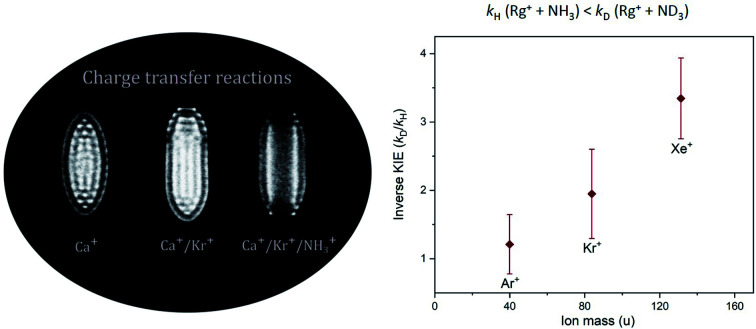

## I. Introduction

Following the detection of CH in the interstellar medium (ISM) in 1937,^1,2^ spectroscopic methods have successfully identified hundreds of different molecular species in interstellar clouds and circumstellar envelopes. Our knowledge of the chemical composition of the ISM is based almost entirely on these spectroscopic observations. However, spectroscopic measurements alone cannot establish how these interstellar molecular species formed. To begin to unravel the complex chemistry of the ISM, a number of astrochemical databases and models have been developed. Some widely-adopted examples include the Kinetic Database for Astrochemistry (KIDA),^3^ the Meudon model for atomic and molecular interstellar gas (known as the Meudon photon-dominated region, PDR, code)^4^ and the UMIST Database for Astrochemistry (UDfA).^5^ While these resources include reaction rate coefficients and branching ratios for many thousands of astrochemically-relevant processes, only a small fraction of these processes have been experimentally examined at temperatures below 300 K. As such, these databases are necessarily reliant on predictions. Rate coefficients for ion-neutral collisions, such as those examined in this work, are often derived from capture theory calculations or extrapolated from measurements taken at room temperature.

Under the extreme conditions prevalent in interstellar clouds—with temperatures spanning 10–100 K, low densities, and the presence of ionising ultraviolet radiation—barrierless reactions between ions and neutral molecules play an important role. Barrierless ion-molecule reactions typically exhibit strong attractive forces between the ion and the dipole (or induced dipole) in the long-range part of the potential. Capture theories account for these long-range attractive forces and assume that the rate-determining step of a reaction is the formation of an intermediate reaction complex, after which products are formed with unit probability. Over the past few decades, capture theories have been found to accurately predict the low-temperature behaviour of a number of ion-molecule reactions.^6^ Indeed, in the absence of experimental data, capture theory models can provide a useful estimate of the rate coefficient for ion-neutral reaction systems. However, as demonstrated in a recent publication,^7^ there are also examples of reaction systems where capture theories fail to adequately describe the likelihood of product formation.

A marked lack of experimental data is evident when considering deuterated reactants; very few experimental studies explicitly consider the reactivity of deuterated species.^8,9^ Deuterated analogues have been found to account for as much as 10% of the total abundance of some molecular species in regions of the ISM, a striking number considering the cosmic ratio of D/H is ≈1.5 × 10^−5^.^10^ This phenomenon, known as deuterium fractionation, cannot be fully accounted for by existing models of interstellar chemistry—highlighting the need for further experimental studies on both hydrogenated and deuterated reaction systems.

In this work, we report four examples of ion-molecule charge transfer reactions where the experimentally-measured rate coefficient is markedly different than that predicted by capture theory models. Charge transfer reactions are studied between ground-state, sympathetically-cooled Kr^+^ or Ar^+^ ions and thermal ammonia isotopologues, NH_3_ or ND_3_, at collision energies <300 K. The reactions are monitored within the environment of Ca^+^ Coulomb crystals, an approach that is ideally suited for such measurements. Both hydrogenated ammonia and fully deuterated ammonia have been observed in the ISM.^11^ While the reactions of heavier rare gas ions are not directly relevant to astrochemistry, lighter rare gas ions are prevalent in the ISM. Of particular relevance to this work, Ar is known to be present in the ISM and Ar^+^ is expected to play a role in interstellar chemical reactions.^12^ As there are few existing experimental studies of charge transfer reactions with rare gas ions, their rate coefficients in astrochemical databases are almost entirely based on capture theory calculations. The findings presented here indicate that capture theory models consistently over-estimate the rate coefficients for charge transfer reactions involving rare gas ions and ammonia molecules.

We measure a significant inverse kinetic isotope effect (KIE), with ND_3_ reacting faster than NH_3_ (*k*_H_/*k*_D_ < 1). Building on our earlier work with Xe^+^ ions, we observe a periodic trend: the magnitude of the inverse KIE shows a dependence on the mass of the rare gas ion. The presence of an inverse KIE cannot be explained within the framework of capture theory models. As the D-substituted bond does not break during the reaction, the systems are described as exhibiting a secondary inverse KIE. The size of the inverse KIE observed for the Kr^+^ + NH_3_ and Kr^+^ + ND_3_ reactions, *k*_H_/*k*_D_ = 0.5, is significant; in systems where secondary KIEs have been previously identified, they are typically minor and range between *k*_H_/*k*_D_ = 0.8–0.9.^13,14^

In a bid to unravel the mechanism responsible for both the rate of charge transfer and the observed trend in the KIE, potential energy surfaces are constructed from *ab initio* calculations. We discuss the importance of certain features of the potential energy surfaces and employ a one-dimensional quantum-mechanical (QM) model to calculate rate coefficients using the most favourable approach of the rare gas ion along the *C*_3_ axis of the ammonia reactant. While the QM model reproduces a number of trends in the experimental measurements, it cannot account for the measured inverse KIEs. We propose tentative explanations for the experimental observations, whilst noting the challenges associated with confirming these theories.

## II. Methods

### A. Experimental methods

**Fig. 1:**
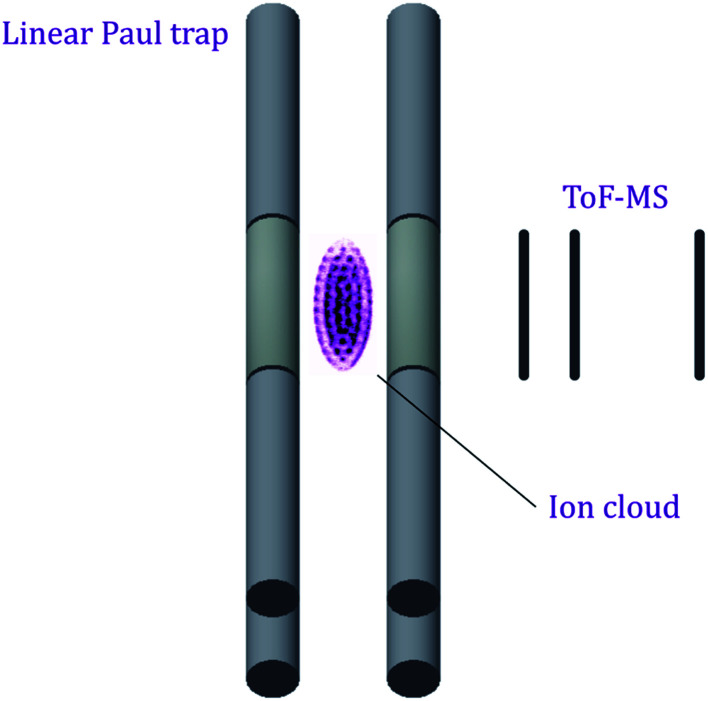
Schematic illustration of the experimental apparatus used to study the charge transfer reactions. The fluorescence emitted by trapped, laser-cooled ions is captured with a charge-coupled device (CCD) camera situated above the ion trap, with an example of a false-colour experimental Ca^+^ Coulomb crystal pictured inside the ion trap (not to scale). Time-of-flight mass spectra (ToF-MS) are recorded by ejecting the ions through a field-free flight tube and onto a microchannel plate (MCP) detector.

The charge exchange reactions take place within the confines of a Ca^+^ Coulomb crystal—a low-density ensemble of laser-cooled ions that adopt a regular three-dimensional spheroidal lattice configuration, with neighbouring ions separated by 10–20 μm.^15^ Ar^+^ or Kr^+^ reactants can be sympathetically cooled into this array of laser-cooled ions, due to efficient elastic collisions with laser-cooled Ca^+^ ions. Translationally-cold trapped Ar^+^ or Kr^+^ ions subsequently undergo reactive collisions with neutral ammonia molecules. A linear Paul trap, depicted schematically in Fig. 1, is used for the confinement and cooling of the ionic species. A detailed description of the ion trap set-up has been given previously.^16–18^

Briefly, a combination of radiofrequency (RF) and static (DC) voltages are applied to the four cylindrical rods of the trap, dynamically trapping the ions. An effusive beam of Ca atoms, produced by a resistively-heated oven, is non-resonantly ionised at the centre of the trap by the frequency-tripled output of a Nd:YAG laser at 355 nm. Ca^+^ Coulomb crystals are formed when a laser cooling scheme addressing 4s^2^S_1/2_ → 4p^2^P_1/2_ (main cooling transition, 397 nm) and 3d^2^D_3/2_ → 4p^2^P_1/2_ (re-pumping transition, 866 nm) is implemented. A CCD camera and a 10× microscope objective lens, located above the ion trap, capture the laser induced fluorescence of the Ca^+^ ions, recording 2-dimensional images of the central slice of the 3-dimensional crystal. Examples of mono- and multi-component crystals recorded in this study are provided in the ESI.†

For the recording of rate coefficients, we follow the protocol described in a previous study involving Xe^+^ charge transfer reactions.^7^ Kr is introduced through a high-precision leak valve, with Kr^+^ ions formed using a (2 + 1) REMPI scheme at 212.6 nm (utilising the tripled output of a Nd:YAG-pumped dye laser). A subset of Kr^+^ ions are produced in the higher energy ^2^P_1/2_ spin state. The ^2^P_1/2_ → ^2^P_3/2_ transition, although electric dipole forbidden, has an Einstein coefficient of *A*_m_ = 2.8 s^−1^ for the magnetic dipole allowed transition, yielding an upper state lifetime of approximately 340 ms.^19^ As a time delay of 30 seconds is introduced following the formation of Kr^+^ ions, this ensures that all Kr^+^ ions are in the ^2^P_3/2_ ground state before the neutral reactants are introduced into the reaction chamber. This delay also gives the Kr^+^ ions time to be sympathetically cooled by elastic collisions with the laser-cooled Ca^+^ ions. For the Ar atoms, a (3 + 1) REMPI scheme is implemented at 314.5 nm (using the doubled output of a Nd:YAG-pumped dye laser). 97 ± 3% of Ar^+^ ions are formed in the ground ^2^P_3/2_ state.^20^ A 30 s delay is again introduced following the formation of Ar^+^ ions to allow for sympathetic cooling to occur.

Room temperature NH_3_ or ND_3_ reactant gases are admitted to the reaction chamber through a second high-precision leak valve, initiating the charge transfer reactions. Partial pressures are measured using an ion gauge calibrated to a residual gas analyser. Systematic uncertainties in pressure gauge readings are difficult to quantify and are not included in our reported uncertainties.^21,22^ Note that this systematic uncertainty does not affect the relative reaction rate coefficients recorded for each reaction system, as we are using the same methodology and ion gauge for each set of experimental measurements (see the ESI† for more details). The progress of the reaction is monitored in two ways, as set out in detail in the ESI.† Quantitative information on the composition of a crystal as a function of the reaction time is established by comparing experimental images with molecular dynamics (MD) simulations, with this analysis complemented by ToF-MS measurements. The presence of any competitive reaction channels is evaluated using the ToF-MS data. A detailed treatment of background reactions (with trace amounts of contaminant gases present in chamber), in addition to the consideration of possible competitive reactions (such as the hydrogen abstraction channels Kr^+^ + NH_3_ → KrH^+^ + NH_2_ and Ar^+^ + NH_3_ → ArH^+^ + NH_2_), can be found in the ESI.† Under our experimental conditions, competing reactions play a negligible role. Any competing or background reactions that do occur are explicitly accounted for in our analysis.

The charge transfer reactions of interest are one-to-one processes (*i.e.*, each reactant ion yields one product ion) and follow pseudo-first order reaction kinetics. As such, an equation of the form [NH_3_^+^]_*t*_ = [Kr^+^]_0_(1 − e^−*k*′*t*^) describes the reaction process, where [Kr^+^]_0_ is the initial number density of Kr^+^ ions and *k*′ is the pseudo-first order reaction rate coefficient. Bimolecular rate coefficients are derived assuming a constant ammonia partial pressure for a given measurement. Tens of repeat measurements are carried out for each charge transfer system, at a number of different ammonia partial pressures (varied between 1 × 10^−9^ and 5 × 10^−9^ mbar). For a more in depth discussion of the measurement of experimental rate coefficients, please see the ESI.†

### B. *Ab initio* calculations

In order to obtain a more detailed picture of the charge transfer reactions, we performed *ab initio* calculations on the two systems following the same approach used for Xe^+^ + NH_3_.^7^ Potential energy surfaces were constructed by means of the multi-configurational self-consistent field method (MCSCF) and the multi-reference configuration interaction (MRCI) method including the Davidson correction (MRCI + Q), as implemented in the MOLPRO quantum chemistry package.^23–25^

The N–H bond length was kept fixed at the NH_3_ equilibrium value of 1.925 *a*_0_, as the equilibrium bond length is similar for NH_3_^+^. We explored the dependence of the potential energy surfaces with respect to the coordinates (*R*, *θ*, *ϕ*, *ρ*), where *R* is the length of the vector **R** describing the position of the rare gas ion (atom) with respect to the center of mass of NH_3_ (NH_3_^+^), *θ* is the angle between the vector **R** and the *C*_3_ axis, *φ* is the angle of rotation of this vector around the *C*_3_ axis, and the angle *ρ* is employed to describe the umbrella motion of ammonia. For NH_3_, the equilibrium value is *ρ* = 112.1° while NH_3_^+^ is planar with *ρ* = 90°.

The ground state of each reaction complex has ^2^A_1_ symmetry and belongs to the *C*_3v_ point group, corresponding to the exit channel Rg(^2^S) + 
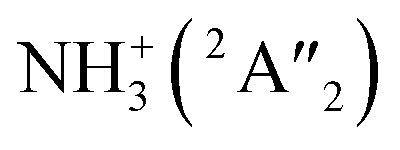
, while the entrance channel Rg^+^(^2^P) + NH_3_(^1^A_1_) gives rise to a ^2^A_1_ state and a doubly-degenerate ^2^E state. All *ab initio* calculations were performed in the abelian subgroups *C*_s_ or *C*_1_. In *C*_s_, the relevant molecular states for the charge transfer process become three ^2^A′ states and one ^2^A′′ state. For the NH_3_-Ar^+^ system, it was necessary to also include the first excited state (^2^E′) of NH_3_^+^ in our calculations as it is close in energy to the Ar^+^ + NH_3_ entrance channel. This leads to two additional states for the complex, ^2^A′ and ^2^A′′, for a total of 4 ^2^A′ and 2 ^2^A′′ states.

The aug-cc-pVTZ basis set is used for all atoms, which is justified by preliminary calculations (see Table SI in the ESI†). In the case of the Kr atom, the 10 innermost electrons were described by an effective core potential (ECP10MDF). The 1s^2^2s^2^2p^6^ orbitals of the Ar atom, the 3s^2^3p^6^3d^10^ orbitals of the Kr atom, as well as the 1s^2^ orbital of the N atom were kept frozen. The active space consists of the remaining orbitals, *i.e.* 3s^2^3p^6^ for Ar, 4s^2^4p^6^ for Kr, 2s^2^p^3^ for N, and 1s for each H atom. In the *C*_s_ point group, this results in (5*a*′ + 1*a*′′) core orbitals for [NH_3_-Ar]^+^ and (7*a*′ + 3*a*′′) core orbitals for [NH_3_-Kr]^+^, with (8*a*′ + 3*a*′′) additional orbitals in the active space for both systems.

**Fig. 2:**
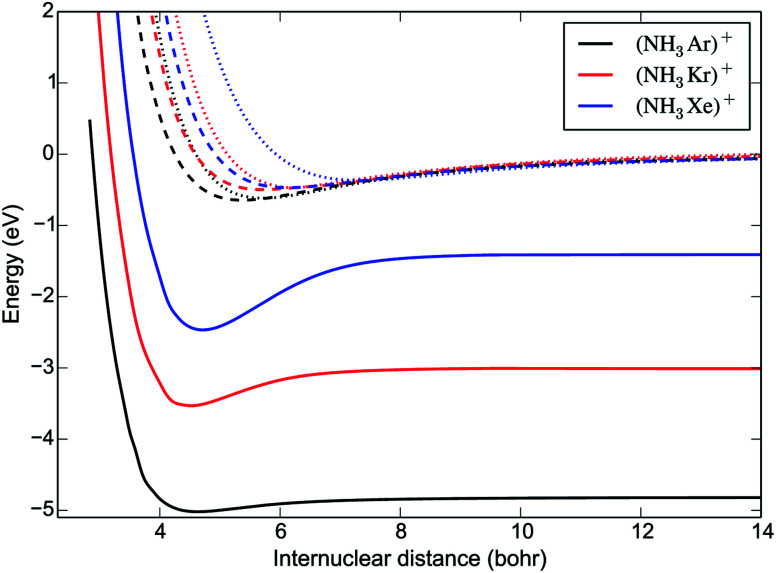
One-dimensional cuts of the PESs of the three ^2^A′ states of the [NH_3_Rg]^+^ complexes relevant to the charge transfer process, as a function of the distance *R* between the Rg^+^ ion and the centre of mass of NH_3_. Solid lines correspond to the ground state PESs dissociating into NH_3_^+^ + Rg(^2^S); dashed and dotted lines correspond to the excited state PESs dissociating into NH_3_ + Rg^+^(^2^P). The zero of energy is set at the entrance channel, NH_3_ + Rg^+^(^2^P). The angles are *θ* = *π* and *ϕ* = 0, corresponding to the Rg^+^ ion approaching the N atom of NH_3_ along the *C*_3_ axis. The umbrella angle is fixed to the equilibrium value of NH_3_, *ρ* = 112°.

As expected, the most favourable orientation for charge transfer is when the Rg^+^ ion approaches the N-atom end of the ammonia molecule along the dipole axis. Fig. 2 compares the potential energy curves (PECs) of the charge transfer channels for the [NH_3_Rg]^+^ systems (including the Rg = Xe results from ref. 7) for this orientation and at an umbrella angle *ρ* = 112°, corresponding to the equilibrium value of NH_3_. The binding energy of the complex increases with the reduced mass, while for the first excited state the opposite trend is observed.

**Fig. 3:**
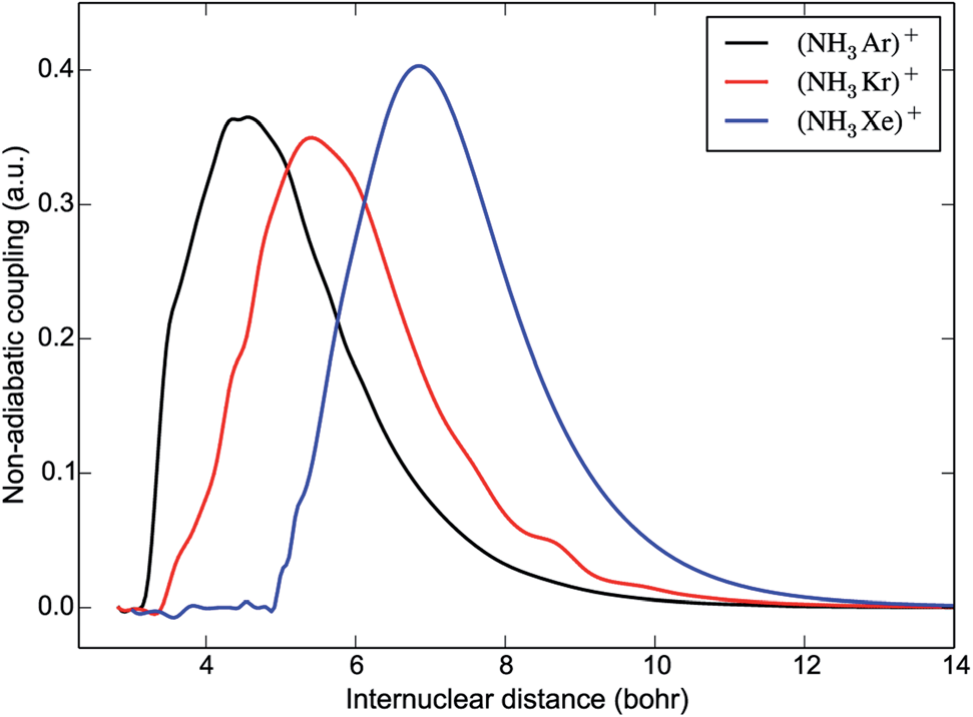
Non-adiabatic coupling between the pairs of reactant and product potential energy curves shown in Fig. 2, for the three different charge transfer systems of interest.

The non-adiabatic couplings were calculated with a three-point central difference method using a displacement parameter *dR* = 0.01 Å. The only non-negligible coupling is between the first two PECs, represented in Fig. 3. The shape of the non-adiabatic coupling is similar for all three systems. It is broad and weak, corresponding to the fact that the PECs do not cross, a situation that can be described by the Rosen-Demkov model of charge transfer.^26^ The maximum of the coupling occurs at shorter distances for the lighter systems. The position of the maximum is at *R* = 4.5 *a*_0_ for [NH_3_Ar]^+^, close to the turning point in the entrance channel. Upon charge transfer, the umbrella angle is relaxed from *ρ* = 112° (the equilibrium value of NH_3_) to *ρ* = 90° (the equilibrium value of NH_3_^+^). However, our calculations show that the non-adiabatic coupling depends only very weakly on the umbrella angle.

## III. Results

### A. Kr^+^ + NH_3_/ND_3_

The experimental rate coefficients measured for the Kr^+^ + NH_3_ and Kr^+^ + ND_3_ reaction systems are *k*_H_ = 0.51(5) × 10^−9^ cm^3^ s^−1^ and *k*_D_ = 1.0(3) × 10^−9^ cm^3^ s^−1^, respectively. The temperatures assigned to the experimental measurements, 246 K for the hydrogenated and 239 K for the deuterated system, represent a weighted average of the temperature of the ammonia reactants (293 K) and the sympathetically-cooled Kr^+^ ions (at ≈13 K, as estimated from MD simulations that account for both the secular motion and the micromotion of the trapped ions).

A substantial inverse KIE is observed, with *k*_H_/*k*_D_ = 0.5(2). The rate coefficients can be compared with the predictions of two capture theory models, namely the average dipole orientation (ADO) model and the adiabatic capture centrifugal sudden approximation (ACCSA). The ADO model is a classical capture theory that describes the interactions between polar molecules and ions, taking into account properties of the neutral polar reactant (such as dipole moment and temperature) and assuming a classical distribution of rotational state populations.^27,28^ ACCSA is a rotationally adiabatic quantum capture theory, developed for symmetric top molecules. It allows the contribution of each rotational state of the neutral species to the overall rate coefficient to be explicitly considered.^29^ While the ACCSA model provides a more complete description of the reaction, ADO is expected to provide a good approximation of rate coefficients at temperatures close to 300 K. As the reactions detailed in this work are conducted at collision energies below 300 K, but under conditions that are still considered “warm” (at around 240 K for the Kr^+^ reactions), both ADO and ACCSA models are employed. Further details on the capture theory calculations are given in the ESI.†

**Fig. 4:**
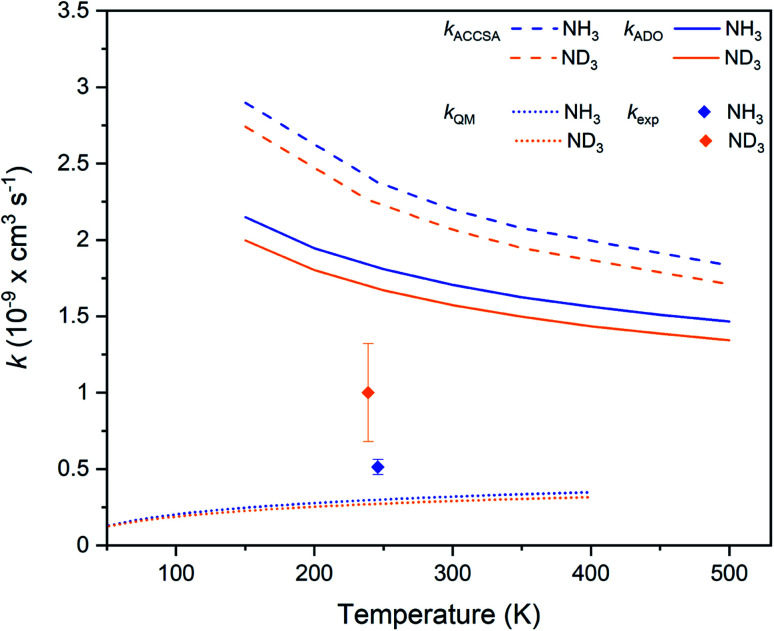
Charge transfer rate coefficients for the Kr^+^ + NH_3_ (blue) and Kr^+^ + ND_3_ (orange) reaction systems, as established from capture theory models (ACCSA and ADO), from a QM model, and from experimental measurements. Error bars indicate the uncertainty in the experimental rate coefficients (see the ESI† for more details).

The experimentally-derived rate coefficients for the two ammonia isotopologues considered in this work are plotted in Fig. 4 as a function of temperature, alongside the calculated ADO and ACCSA rate coefficients. The experimental rate coefficients are considerably lower than predicted by either capture theory model. Additionally, both ADO and ACCSA models predict a weak normal KIE, in contrast to the experimentally-observed inverse KIE. Additional calculations with a method based on the statistical adiabatic channel approach^30,31^ also predict a normal KIE.

To gain more insights into the charge transfer process, we employed a one-dimensional quantum-mechanical (QM) dynamical model corresponding to the most favorable approach along the *C*_3_ axis of NH_3_ represented in Fig. 2. The radial time-independent Schrödinger equation was solved using the close-coupling formalism to obtain reaction cross sections on an energy grid, from which rate coefficients can be calculated.^32,33^ The rate coefficients, as shown in Fig. 4, are seen to be much smaller than those obtained with capture theory. As there is only a weak non-adiabatic coupling between the PECs, not all collisions lead to a reaction. The results are also below the experimental rate coefficients, and predict once again a normal KIE. A limitation of the one-dimensional QM model is that it does not take into account the increase in lifetime that occurs due to intramolecular vibrational redistribution (IVR) between different degrees of freedom of the reaction complex. In the QM model, if charge transfer does not take place within one vibrational period along the reaction co-ordinate, then no products are predicted. However, the effect of intramolecular vibrational redistribution is that the complex is much longer lived than one vibrational period, enhancing the probability that charge transfer occurs in the lifetime of the complex. This explains why the QM rate coefficients are lower than those observed experimentally.

### B. Ar^+^ + NH_3_/ND_3_

**Fig. 5:**
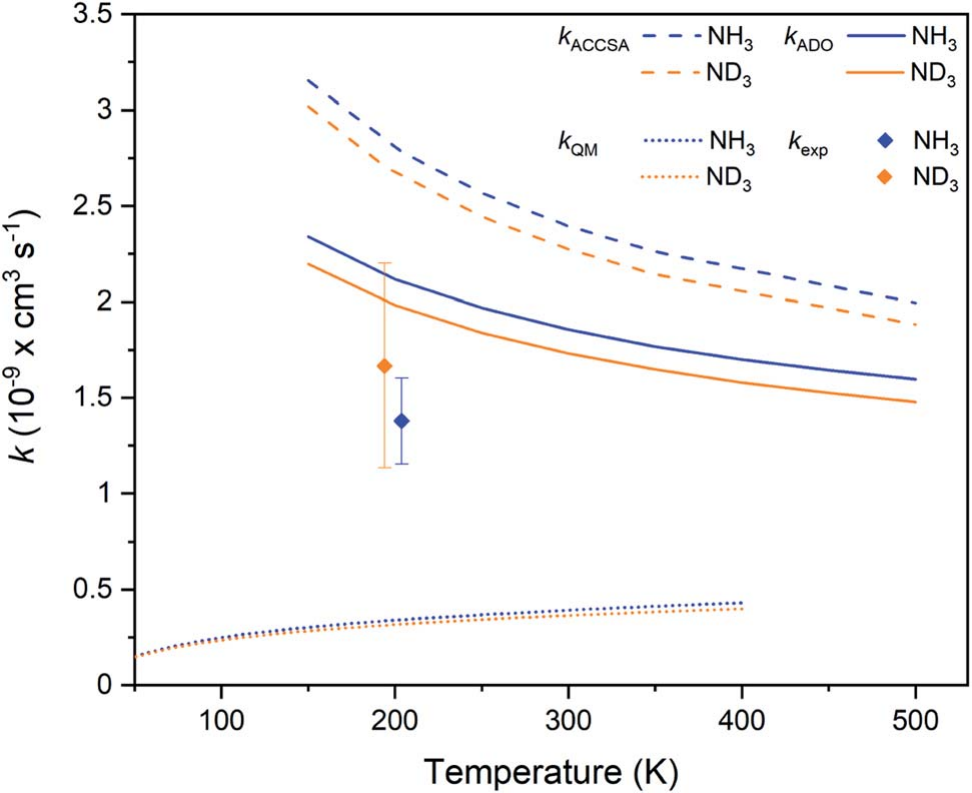
Charge transfer rate coefficients for the Ar^+^ + NH_3_ (blue) and Ar^+^ + ND_3_ (orange) reaction systems, as established from capture theory models (ACCSA and ADO), from a QM model, and from experimental measurements. Error bars indicate the uncertainty in the experimental rate coefficients (see the ESI† for more details).

The experimental rate coefficients for the Ar^+^ + NH_3_ and Ar^+^ + ND_3_ reaction systems are *k*_H_ = 1.4(2) × 10^−9^ cm^3^ s^−1^ and *k*_D_ = 1.7(5) × 10^−9^ cm^3^ s^−1^, respectively. A small inverse KIE is observed, with *k*_H_/*k*_D_ = 0.8(3), although it should be noted that a normal KIE is within the stated uncertainty range. As shown in Fig. 5, the experimental rate coefficients are again suppressed in comparison to the capture theory predictions (although not to the same extent as seen in the Kr^+^ reactions), but are above the QM model predictions. As the mass-to-charge ratio of Ar^+^ ions is approximately equal to that of the laser-cooled Ca^+^ ions, sympathetic cooling is very efficient. Ar^+^ ions are cooled to a temperature of around 3 K (based on MD simulations), yielding a collision energy of 204 K for the hydrogenated and 194 K for the deuterated reaction system. As with the Kr^+^ reactions, details relating to the experimental rate coefficients and capture theory calculations are provided in the ESI.†

## IV. Discussion

**Fig. 6:**
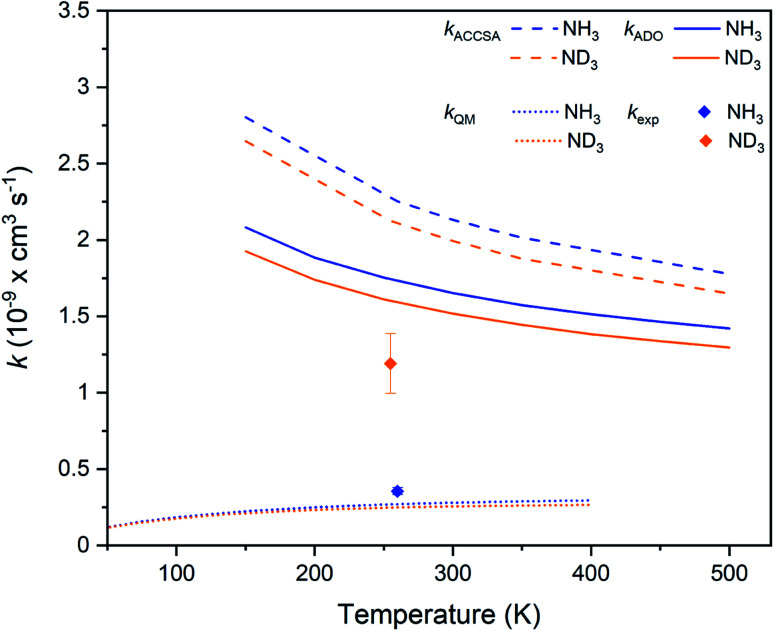
Charge transfer rate coefficients for the Xe^+^ + NH_3_ (blue) and Xe^+^ + ND_3_ (orange) reaction systems, as established from capture theory models (ACCSA and ADO), from a QM model, and from experimental measurements.^7^

Table 1 sets out the experimentally measured and predicted rate coefficients and KIEs for the systems of interest in this work. Details on the properties of the previously investigated Xe^+^ + NH_3_ and Xe^+^ + ND_3_ charge transfer reactions (studied under comparable experimental conditions to this work) are also included, and are plotted in Fig. 6.^7^ Finally, rate coefficients from ion cyclotron resonance (ICR) cell and selected-ion flow tube (SIFT) experiments (carried out at 300 K) are provided in Table 1 for each of the hydrogenated systems.^34,35^

**Table 1:** Experimental, ADO, ACCSA and QM rate coefficients (in cm^3^ s^−1^ × 10^−9^) for the charge transfer reactions between Xe^+^, Kr^+^ or Ar^+^ ions and the two ammonia isotopologues of interest. The experimental uncertainty in the last decimal place is stated in parentheses; for example, *k*_exp_ = (0.36 ± 0.02) × 10^−9^ cm^3^ s^−1^ for the Xe^+^ + NH_3_ system at 260 K (see ESI for details on the calculations). Previous work conducted at 300 K is included for comparison

Reaction system	*k*_exp_	*k*_H_/*k*_D_	*k*_ADO_	*k*_ACCSA_	*k*_QM_	Temperature (K)
Xe^+^ + NH_3_	0.6(1)^a^		1.65	2.13		300
	0.8(2)^b^					
	0.36(2)^c^		1.74	2.25	0.28	260
Xe^+^ + ND_3_	1.2(2)^c^	0.30(5)	1.60	2.12	0.25	255
Kr^+^ + NH_3_	0.8(2)^a^		1.71	2.20		300
	0.7(1)^b^					
	0.51(5)		1.82	2.38	0.34	246
Kr^+^ + ND_3_	1.0(3)	0.5(2)	1.69	2.26	0.30	239
Ar^+^ + NH_3_	1.8(4)^a^		1.86	2.40		300
	1.4(2)		2.11	2.78	0.38	204
Ar^+^ + ND_3_	1.7(5)	0.8(3)	2.00	2.70	0.35	194

a Derai *et al.*^34^

b Giles *et al.*^35^

c Petralia *et al.*^7^

In every instance, the predictions from capture theory models are higher than the experimentally-measured rate coefficients. Of the six reactions considered, only those involving argon ions (Ar^+^ + ND_3_ from this work, and Ar^+^ + NH_3_ recorded using an ICR cell at 300 K) yield rate coefficients that are within the uncertainty range of capture theory predictions—and even then, the (more robust) ACCSA model predicts a higher rate coefficient. The experimental rate coefficients reported in this work and in our previous work on Xe^+^,^7^ recorded at temperatures between 194 K and 260 K, are consistently lower than the rate coefficients reported from ICR cell and SIFT experiments at 300 K. It is, however, difficult to directly compare measurements recorded using ICR or SIFT approaches with those recorded using Coulomb crystals, owing to the different experimental conditions involved (see ESI† for more details).

These findings are consistent with previous work on the He^+^ + NH_3_ system (conducted at 300 K), where the experimental rate coefficient was also found to be significantly lower than the ADO capture theory prediction.^36^ The He^+^ + NH_3_ system is more complex than the reactions considered in this work, as charge transfer is not the dominant reaction channel; NH_2_^+^ + H + He products are preferentially formed, whereas charge transfer is dominant in the Xe^+^ + NH_3_, Kr^+^ + NH_3_ and Ar^+^ + NH_3_ systems.^36^ As the He^+^ + NH_3_ experimental study established the overall rate coefficient for product formation, direct comparison with capture theory predictions was possible (capture theories calculate the probability of a reaction occurring; they cannot discriminate between the formation of different products in systems where more than one reaction channel is energetically open). The rate coefficients included in the KIDA database for the He^+^ + NH_3_ system are entirely based on a classical capture theory model developed by Su & Chesnavich (SC).^37^ The SC model takes into account the dipole moment and polarisability of the neutral species and utilises an empirical formula to yield reaction rate coefficients as a function of temperature. As the SC model over-estimates the experimental He^+^ + NH_3_ reaction rate coefficient at 300 K, it is unlikely to be an appropriate model for describing the low-temperature behaviour of the system.

For reactions that proceed slower than capture theory predictions (*i.e.*, when the formation of a reaction complex does not necessarily yield products), a more nuanced approach needs to be adopted to describe the likelihood of product formation. For example, the presence of a submerged barrier along the reaction coordinate will increase the likelihood that a reaction complex dissociates back to reactants, thereby decreasing the rate coefficient for product formation.^38^ The charge transfer reactions reported in this work are evidently not capture-limited.

The mechanism of electron transfer is not straightforward; no energetically-accessible crossing points have been identified between the reactant and product PECs—not only when considering the two-dimensional cuts through the surfaces as depicted in Fig. 2, but also when considering a wide variety of different *ϕ*, *θ* and *ρ* values. The one-dimensional QM model predicts a rate coefficient that is systematically smaller than the experimental measurements (as well as those obtained with capture theories). In other words, not all collisions are reactive, which can be explained by the weak coupling between the PECs. The QM rate coefficients decrease with increasing mass of the rare gas ion, corresponding to the experimental observations (for charge transfer with NH_3_). The QM model also suggests that the rate coefficients will decrease with decreasing temperature (for all systems), as opposed to the predictions of capture theories. Finally, we note that the QM model predicts that the rate coefficient should be smaller for ND_3_ than for NH_3_, which shows that this model is insufficiently detailed to explain the observed inverse isotope effect. In particular, it appears that the vibrations of the reaction complex are likely to play a role in the charge transfer process.

**Fig. 7:**
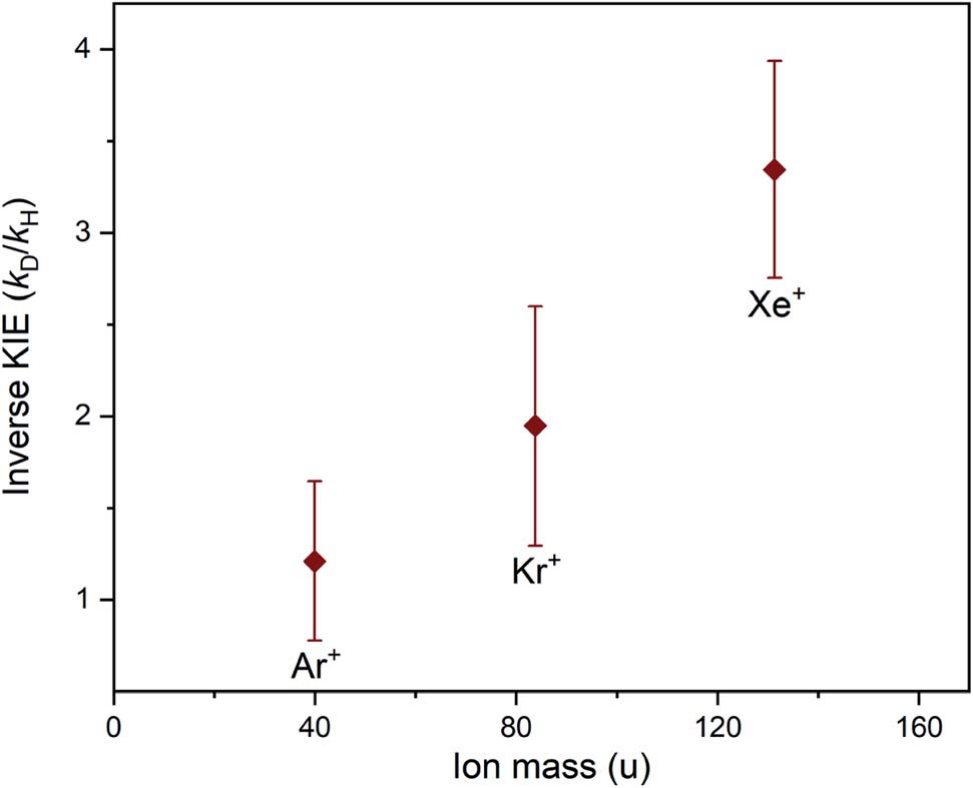
Inverse KIEs observed for the charge transfer reactions between Xe^+^, Kr^+^ or Ar^+^ ions and the two ammonia isotopologues (NH_3_ or ND_3_). The magnitude of the inverse kinetic isotope effect is plotted as a function of the mass of the rare gas ion, showing a mass-dependent trend. The error bars arise from the uncertainty in the experimental rate coefficients. Note that each reaction system was studied at a different temperature (see Table 1).

There is a clear periodic trend in the magnitude of the inverse KIE observed in the charge transfer reactions of Xe^+^, Kr^+^ and Ar^+^ with ammonia. The magnitude of the KIE displays a dependence on the mass of the rare gas ion, with the heaviest (Xe^+^) ion showing the strongest inverse KIE and the lightest (Ar^+^) ion displaying the weakest inverse KIE. The inverse KIE observed for Kr^+^ falls in between the Xe^+^ and Ar^+^ cases. This trend can be clearly identified in Fig. 7, where the KIE is plotted as a function of ionic mass.

There are several steps involved in the transfer of an electron from a neutral ammonia molecule to a rare gas ion, as set out in the general reaction scheme 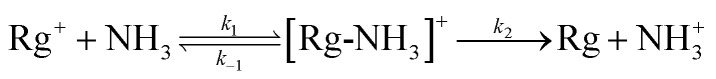, where *k*_1_ represents the rate of capture, *k*_−1_ represents the rate of dissociation of the complex back to reactants, and *k*_2_ represents the rate of charge transfer within the complex and the subsequent formation of products. It is the relative magnitudes of *k*_1_, *k*_−1_ and *k*_2_, related to the properties of the [Rg-NH_3_]^+^ complex, that dictate whether the reaction complex proceeds to product formation, or whether it falls apart to re-form reactants.

For a process that obeys pure capture theory, *k*_2_ ≫ *k*_1_ > *k*_−1_. Complex formation (*k*_1_) is the rate-limiting step; with *k*_2_ being much larger than *k*_−1_, every captured complex proceeds to products. In the systems examined in this work, we deduce that the rate of charge transfer (*k*_2_) is not significantly larger than *k*_1_ and *k*_−1_ (and decreases along the series from Ar^+^ to Xe^+^). Hence the experimental rate coefficients for charge transfer are lower than capture theory predicts. The key factor then becomes the competition between *k*_2_, product formation, and *k*_−1_, dissociation of the complex back to reactants.

It is likely that *k*_−1_ is only very weakly dependent on the mass of the rare gas ion, as *k*_−1_ will predominantly depend on the vibrational motion of the ammonia entity. To first order, one could approximate *k*_−1_ as having a magnitude comparable to the vibrational frequency along *R* for a simple atom-ion collision. However, here we are dealing with a polyatomic species; we also need to consider that the lifetime of the reaction complex may be extended by intramolecular energy transfer, which has the effect of lowering *k*_−1_. There is a clear isotope effect here, arising from the very different vibrational frequencies associated with the deuterated and hydrogenated ammonia moieties in the reaction complex. There is a greater density of states in the [Rg-ND_3_]^+^ complex compared to the [Rg-NH_3_]^+^ complex, resulting in an increased lifetime of the complex and hence a lower *k*_−1_ for the deuterated compounds. This, in turn, sees a greater proportion of [Rg-ND_3_]^+^ complexes proceed to products, yielding a faster rate coefficient for charge transfer when compared to the [Rg-NH_3_]^+^ system. On changing the rare-gas ion, the magnitude of *k*_2_ changes. For example, we expect *k*_2_ to be greatest for Ar^+^ when compared to Kr^+^ and Xe^+^ (as discussed below). With a greater *k*_2_, the difference between *k*_−1_ for ND_3_ and NH_3_ has a less important effect on the overall rate coefficient and therefore the inverse kinetic isotope effect is less marked.

It is exceedingly challenging to experimentally probe which of the competing processes (*k*_1_, *k*_−1_ or *k*_2_) is primarily responsible for the observed inverse KIEs. It is also challenging to employ more sophisticated theoretical methods to describe the charge transfer reaction systems. While theoretical methods such as flexible microcanonical transition state theory^39^ could allow for the inclusion of IVR when calculating the lifetime of a reaction complex, this approach was developed for processes occurring on a single PES. In this work, multiple PESs and non-adiabatic couplings of high dimensionality need to be considered, since our results indicate that the vibrational modes of ammonia play a role. The difficulty of computing such PESs and couplings also limits the possibility of running trajectory calculations to establish theoretical predictions for the relative importance of the competing processes at play. We can, however, examine the feasibility of the above mechanism based on the features of the calculated PECs.

While the rate coefficient for charge transfer is related to the strength of the coupling between the PECs (as depicted in Fig. 3), it is difficult to precisely predict the probability of charge transfer based on this property alone. Other factors—such as the width and location of the non-adiabatic coupling, in addition to the shape of the underlying surfaces, their energetic separation, and the collision energy—are also important when considering the likelihood of charge transfer. The location of the maximum of the coupling strength near the inner turning point of the one-dimensional entrance-channel potential is (amongst other factors) likely to contribute to faster charge transfer, and hence a larger value for *k*_2_, in the case of Ar^+^. As noted above, the increase in the density of vibrational states for [Rg-ND_3_]^+^ will also extend the lifetime of the complex compared to the [Rg-NH_3_]^+^ system; with more degrees of freedom, the available energy could be dispersed into more modes and therefore take longer to localise in the mode corresponding to dissociation (reforming reactants), lowering *k*_−1_ and allowing more time for product formation to occur. This explanation is consistent with the experimentally observed periodic trend. The Ar^+^ system displays the lowest inverse KIE, and also features the complexes with the lowest relative densities of states; the [Xe-ND_3_]^+^ complex has the highest density of states, and the Xe^+^ system exhibits the highest inverse KIE.

A previous theory study of secondary KIEs broadly supports this proposed explanation. Glad *et al.* found the structure and properties of the transition state complex determined whether a normal or inverse KIE was expected for a series of S_N_2 reactions (it should be noted that only comparatively small inverse or normal KIEs were predicted, ranging from 0.9–1.1).^14^ From a consideration of the various factors that might contribute to KIEs, the study concluded that the vibrational degrees of freedom of the reaction complex were primarily responsible for any overall inverse KIEs.

## V. Conclusions

Rate coefficients for the charge transfer between two isotopologues of ammonia, NH_3_ and ND_3_, and two rare gas ions, Kr^+^ and Ar^+^, have been measured. An inverse KIE is evident, where ND_3_ reacts faster than NH_3_. These findings build upon an earlier study, where we identified a significant KIE in the charge transfer of Xe^+^ with ammonia isotopologues.^7^ Secondary inverse KIEs are relatively uncommon, having only been identified in a handful of previous studies. In systems where secondary inverse KIEs have been seen, they are typically modest effects (with *k*_H_/*k*_D_ = 0.8–0.9).^13,14^ In contrast, the inverse KIEs we observe in the charge transfer reactions of rare gas ions with ammonia isotopologues range in magnitude from 0.3 (for Xe^+^) to 0.8 (for Ar^+^).

In all of the charge transfer systems examined in this work, capture theories overestimate the experimental rate coefficients. Capture theories cannot account for the presence of any inverse KIEs. We postulate that capture theories fail to describe the reaction kinetics in these systems because the electron transfer process is not capture limited; as there is no energetically-accessible crossing point between the reactant and product PECs, the charge transfer process is far from straightforward. The non-adiabatic coupling between the reactant and product PECs is broad and weak, and the one-dimensional QM model predicts a lower rate coefficient than is experimentally observed. The QM model also fails to account for the observed inverse KIEs. We suggest that the properties of the [Rg-ammonia]^+^ reaction complex, including the density of vibrational states and the expected lifetime of the complex, will play a key role in influencing the likelihood of a given reaction proceeding to products. While this explanation could account for the observed periodic trend in the inverse KIE, further experimental and theoretical work is needed to confirm the validity of this theory.

Capture theory predictions of reactivity are frequently incorporated into astrochemical databases in situations where there are no experimental measurements available. As the findings of this work illustrate, capture theories are not always accurate at predicting reaction rate coefficients—even for seemingly simple charge transfer processes involving rare gas ions. Further work is needed to gain insights into when capture theory predictions can be confidently utilised.

## Data availability

Supporting data can be obtained from the Oxford Research Archive.

## Author contributions

A. T., K. P. and P. B. performed the Ar^+^ experiments. A. T. and P. B. performed the Kr^+^ experiments. A. T. analysed the experimental data and calculated the ADO rate coefficients. K. P. wrote the code for the calculation of the ACCSA rate coefficients. B. R. H. devised the project, supervised the experiments, and oversaw the data analysis and capture theory calculations. T. P. S. and B. R. H. interpreted the results. J. L. performed the *ab initio* calculations, constructed the potential energy surfaces, and devised the one-dimensional quantum-mechanical model. All authors contributed to writing the paper.

## Conflicts of interest

There are no conflicts to declare.

## Supplementary Material

SC-012-D1SC01652K-s001
