# Supplementary material for: Inverse kinetic isotope effects in the charge transfer reactions of ammonia with rare gas ions
Source: Chem Sci. 2021 Jun 22;12(29):10005–13. doi: 10.1039/d1sc01652k (PMC8317658; doi:10.1039/d1sc01652k)
Supplement: SC-012-D1SC01652K-s001 [file SC-012-D1SC01652K-s001.pdf]

Electronic Supplementary Material (ESI) for Chemical Science.
This journal is © The Royal Society of Chemistry 2021

Supplementary Material: Inverse kinetic isotope effects in the charge transfer reactions of ammonia with rare gas ions

(Dated: June 11, 2021)

Supplementary note 1. Monitoring the charge transfer reactions

In Figures 1 (a) and (b), Coulomb crystal images are provided at different reaction times. These snapshots show the progress of charge transfer reactions between Kr^+ or Ar^+ ions and neutral ammonia molecules. With Ar^+ and Ca^+ exhibiting very similar mass-to-charge ratios (both species are singly charged with a mass of 40 u), they experience similar trapping forces and so are expected to be located at approximately the same distance from the trap axis. As the cooling lasers are applied from one direction, radiation pressure induces a spatial separation between the equilibrium positions of Ca^+ and Ar^+ ions in the crystal: Ar^+ ions are preferentially located at the top of the crystal (in the orientation depicted in Figure 1). The number of ions of each species within a Coulomb crystal at a given moment in time is established by comparing the experimental images with images produced by a custom-written molecular dynamics (MD) simulation software package. Iterative MD simulations of the crystal are undertaken to determine ion numbers for every time point on the exponential growth curve (see Figure 2b), for every reaction measurement undertaken.

A detailed description of the comparison method can be found in previous work.[1] Figures 1 (c) and (d) show the MD images that correspond to each experimental image above, where the non-fluorescing reactant species are shown in colour. The accuracy with which we can establish the number of ions, under our experimental conditions, is dependent on the species. We have an uncertainty range of ±10 ions for Ca^+, ±20 for Kr^+, ±40 for Ar^+ and ±5 for ${NH_3}^+$ and ${ND_3}^+$.

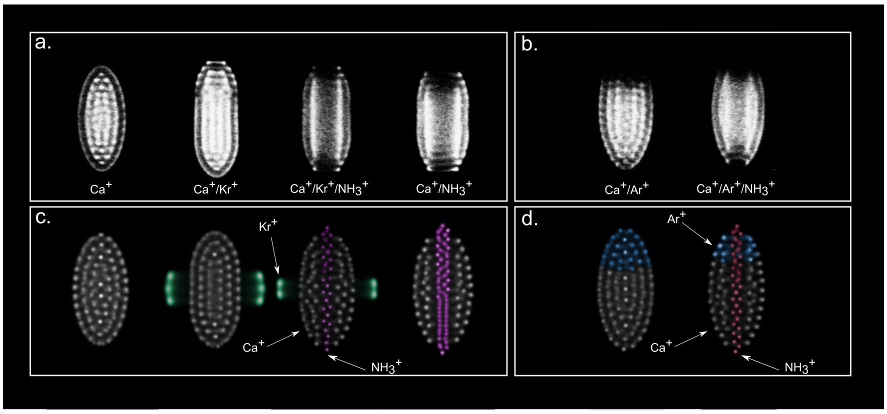

FIG. 1: (a) From left to right: Experimental images of a Ca^+ Coulomb crystal, a bi-component Ca^+ and Kr^+ crystal, a tri-component crystal midway through the charge transfer reaction, and a crystal at the completion of the reaction (i.e., when no Kr^+ reactants remain). (b) Experimental Coulomb crystal images at the start and midway through a charge transfer reaction with Ar^+ ions. (c) and (d) MD simulations corresponding to the experimental Coulomb crystal images depicted in panels (a) and (b), above. The positions of the non-fluorescing co-trapped ions are explicitly shown in the simulations alongside the fluorescing Ca^+ ions. While these non-laser-cooled species are not visible in the experimental images, the positions and numbers of co-trapped species can be established from the MD simulations.

Supplementary note 2. Competitive reaction pathways

The energy of the various possible products obtained with basis sets of increasing size is presented in Table I. For the reactions of NH_3 with Xe^+, only the charge transfer channel is exothermic, as established previously.[1] For the reactions with Ar^+ or Kr^+, the proton transfer channel is also exothermic, leading to products NH_2 + ArH^+ or KrH^+. For Ar^+, the channel leading to the formation of NH_2^+ + H + Ar is predicted to be exothermic with the CI method, but endothermic with the CI+Q method. However, these reactions require bond-breaking and are not observed in the present experiment.

TABLE I: Energy of the possible products from the reaction NH_3 + Rg^+ with the MRCI method and the MRCI + Davidson correction (CI+Q) and various basis sets, in eV, following geometry optimization. The origin of energies corresponds to the energy of the reactants.

	MRCI			MRCI+Q		
	AVTZ	AVQZ	AV5Z	AVTZ	AVQZ	AV5Z
NH_3^+ + Ar	-5.693	-5.776	-5.802	-5.430	-5.519	-5.544
NH_2 + ArH^+	-1.782	-1.865	-1.905	-1.256	-1.311	-1.343
NH_2^+ + H + Ar	-0.202	-0.263	-0.282	0.406	0.369	0.358
NH_3^+ + Kr	-3.658	-3.690	-3.724	-3.804	-3.875	-3.914
NH_2 + KrH^+	-0.527	-0.585	-0.605	-0.151	-0.182	-0.197
NH_2^+ + H + Kr	1.587	1.546	1.518	2.055	2.031	2.005
NH_3^+ + Xe	-1.974	-1.924	-1.913	-2.110	-2.055	-2.043
NH_2 + XeH^+	0.480	0.421	0.364	0.850	0.804	0.746
NH_2^+ + H + Xe	4.314	3.370	2.930	4.881	3.852	3.366

As in the analysis of the Xe^+ + NH_3 and ND_3 reaction systems, very few background reactions were observed on the timescale of the charge transfer reactions. Those that did occur, mainly with trace amounts of background H_2O in the chamber, were explicitly accounted for in our analysis. This protocol has been outlined in an earlier publication.[1] Previous studies on the Kr^+ + NH_3 reaction system at 300 K found electron transfer to be the dominant reaction pathway, with the competitive hydrogen abstraction channel, $Kr^+ + NH_3 \longrightarrow KrH^+ + NH_2$, established as having a branching ratio of less than 1% — equivalent to the reported uncertainty of the study.[2] Under our experimental conditions, at around 240 K, no KrH^+ or KrD^+ ions are detected in the ToF-MS data; the hydrogen abstraction channel is either entirely absent, or it plays such a negligible role that it falls within our experimental uncertainty. As such, in our analysis we assume that every Kr^+ that reacts produces charge transfer product ions ($NH_3{}^+$ or $ND_3{}^+$).

A more nuanced consideration of competitive reaction channels is required for the Ar^+ system. Previous experimental studies have identified three pathways following reactive Ar^+ + NH_3 collisions at 300 K,[2]

$$Ar^+(^2P_{3/2}) + NH_3(^1A_1) \longrightarrow Ar + NH_3{}^+(^2A_2'') \quad (1)$$

$$Ar + NH_3{}^+(^2E) \longrightarrow Ar + H + NH_2{}^+ \quad (2)$$

$$ArH^+ + NH_2 \quad (3)$$

with reported branching ratios of 0.87, 0.07 and 0.06 (respectively). Reaction 1, the charge transfer process, has an exothermicity of $\Delta H = -5.60$ eV.[3] There is a discrepancy in the literature relating to the energy of the first electronically excited state of $NH_3{}^+$. The experimental work of Derai *et al.* place this channel approximately 4.7 eV above the energy of ground state $NH_3{}^+$, making it accessible at 300 K. They observe $NH_2{}^+$ products and attribute these to reaction 2: the dissociation of electronically excited $NH_3{}^+$ ions. The MRCI+Q calculations carried out as part of this work, in agreement with an earlier study that reported the adiabatic excitation energy to be approximately 6 eV,[4] suggest that the electronically excited state of $NH_3{}^+$ is inaccessible at 300 K. While attempting to explain this discrepancy is beyond the scope of this work, we note the disagreement between the photoelectron spectroscopic measurements and two independent sets of theory calculations. We observe no $NH_2{}^+$ products in our ToF-MS measurements, suggesting that the formation of excited state $NH_3{}^+$ (channel 2) is either energetically closed under our experimental conditions (at 204 K)—as predicted by the theory work—or is such a minor channel that it falls under our detection sensitivity level. The ToF-MS data also show no evidence of ArH^+ ions within our measurement resolution. While this channel is energetically open, the lack of any ArH^+ ions in the ToF-MS traces indicates that the branching ratio of the hydrogen abstraction channel (reaction 3) is < 0.05 under our experimental conditions.

Even if a small number of ArH^+ product ions are formed, these do not affect the calculation of bimolecular rate constants for the charge transfer reaction. Only product ions that are formed in the dark core—i.e., species that have a mass-to-charge ratio below 40 u, which excludes any possible ArH^+products—are included in our analysis. We are not aware of any previous studies on the Ar^+ + ND_3 reaction system. We do not detect any evidence of deuterium abstraction products, ArD^+, within our experimental uncertainty, indicating that this is again a minor channel with a branching ratio of < 0.05 under our experimental conditions.

Supplementary note 3. Trapping efficiency

Table II summarises the trap depths experienced by each ionic species involved in the charge transfer reactions. More information regarding the calculation of trap depths can be found elsewhere.[1] The exothermicities of the charge transfer reactions, $\Delta H = -3.84$ eV and $\Delta H = -5.60$ eV for the Kr^+ and Ar^+ reaction systems, respectively,[3] are significantly lower than the trap depths experienced by the ${NH_3}^+$ and ${ND_3}^+$ product ions. Moreover, the sympathetic cooling forces act on the ammonia ions immediately after their formation to dissipate any kinetic energy. We therefore assume that all product ions are confined by the trapping potential.

TABLE II: Trap depths experienced by the species involved in the charge transfer reactions.

Ion	Ca^+	Kr^+	Ar^+	${NH_3}^+$	${ND_3}^+$
Trap depth (eV)	2.9	1.0	2.9	7.5	6.3

Supplementary note 4. Reaction rate coefficients

The kinetic model used to calculate reaction rate coefficients has been derived and described in detail in a previous publication.[1] Briefly, a pseudo-first order rate coefficient, k', is calculated by plotting the number of ${NH_3}^+$ ions established from the comparison with MD simulations as a function of time (see Figure 2b). The data points are fit to an exponential growth curve that follows the equation

$$[{NH_3}^+]_t = [Kr^+]_0 \left(1 - e^{-k't}\right), \tag{1}$$

where $[Kr^+]_0$ represents the initial number density of the rare-gas ions and $[{NH_3}^+]_t$ the number density of the product ammonia ions as a function of time. k' is given by

$$k' = k[NH_3], \tag{2}$$

where $[NH_3]$ is the partial pressure of the ammonia reactants and k is the bimolecular rate coefficient.

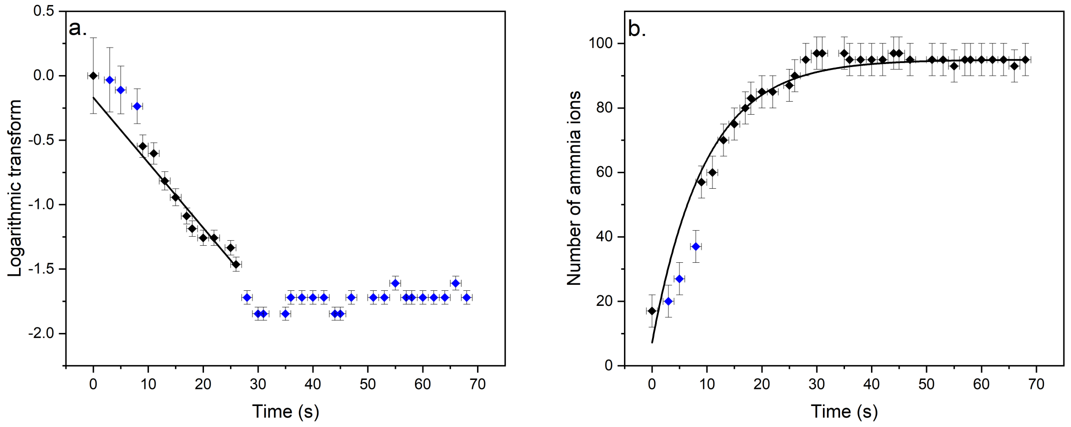

FIG. 2: (a) Linear form of eq. (1). The first three points (after the reaction is initiated) deviate from the linear fit, as they were recorded before the pressure stabilised. All black data points are included in the fit to ascertain k'. Data points recorded after approximately 28 seconds appear as a horizontal line as the reaction has gone to completion; the number of ammonia ions is no longer changing. (b) Exponential growth fit to eq. (1), taking into account the data points recorded once conditions were stable. Error bars of $E_y = \pm 5$ ions and $E_x = \pm 1$ s account for the accuracy of the MD simulations and experimental uncertainty in the reaction time, respectively.

Due to the non-zero time taken for the ammonia partial pressure to reach the desired value, the first few data points recorded for each reaction do not fit well to the exponential growth model of eq. (1). To establish when

uniform conditions have been achieved, the data are fit to a linear form of eq. (1). Points that clearly deviate from the linear fit at early reaction times are discarded. As illustrated in Figure 2a, the linear fit facilitates the easier identification of these points. However, it is used only to establish when the partial pressure of ammonia has become stable, with the exponential form of the graph (Figure 2b) used for the calculation of the pseudo-first order rate coefficients. The resulting pseudo-first order rate coefficients, k', are set out in Table III, with the reported uncertainty derived from the standard error associated with the fit of eq. (1) to the experimental data points.

A bimolecular rate coefficient, k, is calculated for each reaction measurement using eq. (2). The k values reported in the manuscript represent the average of all rate coefficients calculated (i.e., considering the values established for every repeat measurement undertaken, for every set of conditions considered, for each of the reaction systems). Uncertainties are stated as the statistical error (one standard deviation). To confirm the validity of this approach, bimolecular rate coefficients are also calculated by plotting the pseudo-first order rate coefficients (k') as a function of the partial pressure of ammonia. Figure 3 illustrates the linear correlation between partial pressure and k', with the gradient of the line corresponding to the bimolecular rate coefficient. While there is excellent agreement between the k values obtained from the two approaches (see Table III), there is a higher uncertainty associated with the rate coefficients obtained from the averaging method. As we have a limited ability to vary the partial pressure of the ammonia reactants (between 1×10^{-9} and 5×10^{-9} mbar), we include the linear fit method simply as a complementary way of examining the data. The rate coefficients obtained from both methods are reported to provide confirmation that our findings are robust. Only rate coefficients calculated from the averaging method are included in the main text.

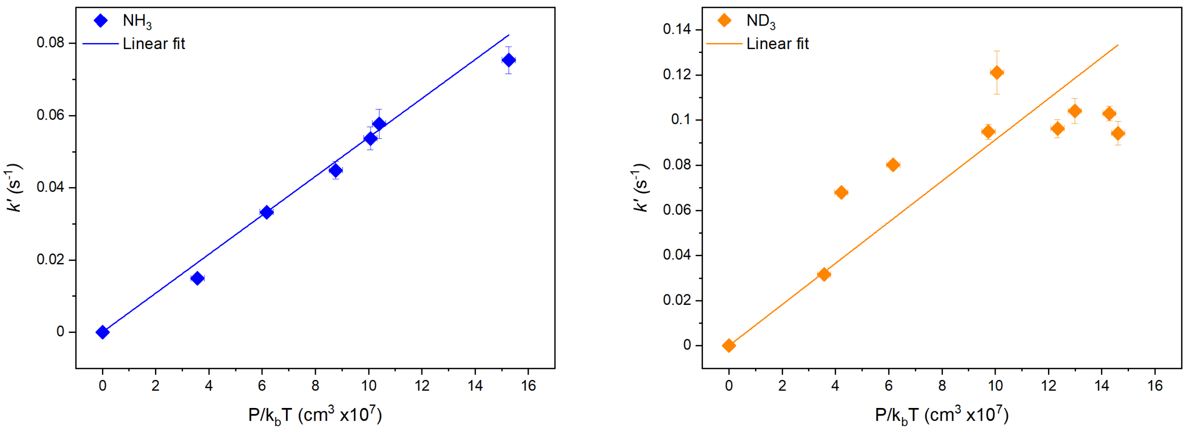

FIG. 3: Pseudo-first order rate coefficients calculated for the Kr^+ reactions are plotted as a function of ammonia neutral reactant partial pressure. The gradient of the linear fit corresponds to the bimolecular rate coefficient, k_{lin}.

TABLE III: Pseudo-first order, k', and bimolecular rate coefficients calculated for the reaction systems of interest using the average, k, and linear correlation, k_{lin}, methods.

	k' ($\times 10^{-2}$ s^{-1})	k ($\times 10^{-9}$ cm^3 s^{-1})	k_{lin} ($\times 10^{-9}$ cm^3 s^{-1})
$Kr^+ + NH_3$	4.7(2)	0.51(5)	0.54(2)
$Kr^+ + ND_3$	8.8(4)	1.0(3)	0.9(1)
$Ar^+ + NH_3$	9.0(8)	1.4(2)	1.3(1)
$Ar^+ + ND_3$	12(1)	1.7(5)	1.5(2)

Before changing to a different reactant species, the reaction chamber is baked for several days and the chamber is pumped down for at least a week. A residual gas analyser (RGA) is employed to ensure that there are no trace amounts of ammonia left in the chamber prior to embarking on a new set of experimental measurements. The partial pressures of ammonia admitted to the reaction chamber are established using an ion gauge, as is typical in Coulomb crystal reaction studies. Despite using the RGA to calibrate the ion gauge, we cannot rule out systematic uncertainties associated with the use of the ion gauge readings. It is difficult to quantify the magnitude of any possible systematic

uncertainties associated with the ion gauge readings. All measurements are undertaken using the same ion gauge and under comparable experimental conditions—enabling us to directly compare the findings with confidence. Additional systematic uncertainties arising from the pressure gauge readings should be considered when comparing the rate coefficients reported in this work to measurements undertaken using different techniques.

When comparing findings reported in this work (and in other similar Coulomb crystal-based studies), it is important to consider the differences in the experimental conditions used. For example, while the ammonia reactants in this work are thermal, the ionic reactants are effectively stationary (i.e., the reactants are not in thermal equilibrium). The experiments in this work are also carried out under ultra-high vacuum conditions (on the order of 1×10^{-9} mbar), in what has been described as a nearly perturbation-free environment.[5,6] In comparison, for example, selected ion flow tube (SIFT) measurements are typically carried out in a helium buffer gas, at pressures that are many orders of magnitude higher (approximately 0.7 mbar).[7] It is therefore difficult to directly compare findings reported from these different experimental methods.

Supplementary note 5. Capture theory rate coefficients

We compare the experimental reaction rate coefficients with predictions from average dipole orientation (ADO) theory[8,9] and adiabatic capture centrifugal sudden approximation (ACCSA) theory.[10] Details on how ADO rate coefficients are calculated have been given elsewhere.[1] ACCSA theory is a rotationally adiabatic quantum capture model that takes into account the quantum numbers (J, j, k, Ω), where J is the total angular momentum, j the molecular angular momentum, K the projection of j onto the molecular-fixed axis, and Ω the projection of J and j onto the body-fixed axis. The ACCSA rate coefficients are calculated using

$$k_{\mathrm{ACCSA}} = \frac{\sum\limits_{jK} g_{jK} \exp\left[-E_{jK}/(k_B T)\right] k_{jK}(T)}{\sum\limits_{jK} g_{jK} \exp\left[-E_{jK}/(k_B T)\right]}, \tag{3}$$

where g_{jK} is the degeneracy factor, E_{jK} the barrier height of the potential curve and k_{jK} the rate coefficient for each rotational state (j, K). Table IV summarises the g_{jK} factors for symmetric top molecules, such as the ammonia, as a function of the rotational state K.[11,12]

K	0	1	2	3	...	$K \not\equiv 0 \pmod 3$	$K \equiv 0 \pmod 3$
g_{jK}	1	2	2	4	...	2	4

TABLE IV: Degeneracy factors, g_{jK}, as a function of K. If K is divisible by 3, $g_{jK} = 4$, otherwise $g_{jK} = 2$.

Rate coefficients for individual rotational states, k_{jK}, are calculated using the equation

$$k_{jK}(T) = \sqrt{\frac{8}{\pi\mu(k_B T)^3}} \int_0^\infty \sigma(j, K, E) E \exp[-E/(k_B T)] dE, \tag{4}$$

where k_B is the Boltzmann constant, E the collision energy, μ the reduced mass, and $\sigma(j, K, E)$ the reaction cross section. The latter is calculated by

$$\bar{\sigma}(j, K, E) = \frac{\pi}{k^2(2j+1)} \sum_{\Omega} \left[J_{\max}(j, K, \Omega) + 1\right]^2, \tag{5}$$

where $k^2 = 2\mu E/\hbar^2$ and $J_{\max}(j, K, \Omega)$ is the maximum angular momentum for which the collision energy is greater than the maximum potential ($E \geq V_{J_{jK\Omega}}(R)$). Figure 4 illustrates the calculation of $J_{\max}$ for the the NH_3 and Ar^+ reaction system at 206 K. $J_{\max} = 51$ is the maximum J value where $E \geq V_{J_{jK\Omega}}(R)$ for every value of R.

To obtain the potential curves, $V_{J_{jK\Omega}}(R)$, one can use

$$V_{JjK\Omega}(R) = \varepsilon_{jK\Omega}(R) + \frac{\hbar^2 J(J+1)}{2\mu R^2} - E_{jK}, \tag{6}$$

where $\varepsilon_{jK\Omega}$ is the adiabatic potential and $E_{jK} = Bj(j+1) + (A-B)K^2$. The rotational constants A and B are 6.19600 cm^{-1} and 9.44430 cm^{-1} for NH_3; 3.14420 cm^{-1} and 5.14280 cm^{-1} for ND_3. $\varepsilon_{jK\Omega}(R)$ is calculated by diagonalisation

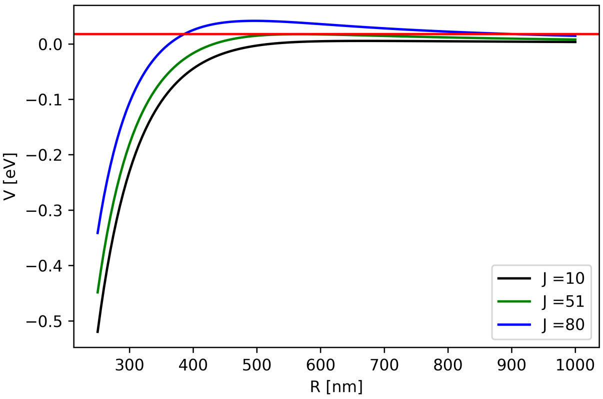

FIG. 4: Potential curves of the $(j, K, \Omega) = (10, 5, 2)$ state with different J values for the NH_3 and Ar^+ reaction system at 206 K. The red line indicates the collision energy, E.

of the coupling matrices $V_{jj'}$ as a function of the distance R between the ammonia and rare gas ion. It is given by

$$V_{jj'} = \left\{ E_{jK} + \frac{\left[j(j+1)\hbar^2 - 2\Omega^2\hbar^2\right]}{2\mu R^2} - \frac{\alpha q^2}{2R^4} \right\} \delta_{ji'} - \frac{q\mu_D \left\{ C \begin{pmatrix} j & j' & 1 \\ -K & K & 0 \end{pmatrix} C \begin{pmatrix} j & j' & 1 \\ -\Omega & \Omega & 0 \end{pmatrix} \left[(2j+1)(2j'+1)\right]^{1/2} \right\}}{3R^2}. \tag{7}$$

μ_D is the dipole moment of the molecule, q the charge of the ion, α the polarisability, $\hbar$ the Plank constant, and C the Clebsch-Gordon coefficients. Note that a matrix size of 201×201 is used for the coupling matrices. The distance between the coupling elements ranges from 1.5 Å to 55 Å with an interval of 0.05 Å.

References

[1] L. Petralia, A. Tsikritea, J. Loreau, T. Softley, and B. Heazlewood, Nature Communications **11**, 1 (2020).
[2] R. Derai, G. Mauclaire, and R. Marx, Chemical Physics Letters **86**, 275 (1982).
[3] P. R. Kemper, M. T. Bowers, D. C. Parent, G. Mauclaire, R. Derai, and R. Marx, The Journal of Chemical Physics **79**, 160 (1983).
[4] A. Viel, W. Eisfeld, S. Neumann, W. Domcke, and U. Manthe, The Journal of Chemical Physics **124**, 214306 (2006).
[5] M. Germann, X. Tong, and S. Willitsch, Nature Physics **10**, 820 (2014).
[6] B. R. Heazlewood, Molecular Physics pp. 1–8 (2019).
[7] K. Giles, N. Adams, and D. Smith, Journal of Physics B: Atomic, Molecular and Optical Physics **22**, 873 (1989).
[8] T. Su and M. T. Bowers, The Journal of Chemical Physics **58**, 3027 (1973).
[9] T. Su and M. T. Bowers, International Journal of Mass Spectrometry and ion processes **17**, 211 (1975).
[10] D. Clary, Molecular Physics **54**, 605 (1985).
[11] G. Herzberg, *Molecular spectra and molecular structure. II. Infrared and Raman spectra of polyatomic molecules*, Molecular Spectra and Molecular Structure (Van Nostrand, 1939).
[12] T. Stoecklin, D. C. Clary, and A. Palma, J. Chem. Soc., Faraday Trans. **88**, 901 (1992).
